# Menopause Rating Scale (MRS) in the Malay language-translation and validation in a multiethnic population of Selangor, Malaysia

**DOI:** 10.1186/s12905-022-01922-8

**Published:** 2022-08-17

**Authors:** Sashimalar Mathialagan, Shamala Ramasamy, Kavitha Nagandla, Wei Fern Siew, Chandrashekhar T. Sreeramareddy

**Affiliations:** 1 School of Postgraduate Studies, International Medical University, Kuala Lumpur, Malaysia; 2 Department of Psychology, International Medical University, Kuala Lumpur, Malaysia; 3 Division of Obstetrics and Gynecology, International Medical University, Kuala Lumpur, Malaysia; 4 Division of Community Medicine, International Medical University, Jalan Jalil Perkasa 19, Bukit Jalil, 57000 Kuala Lumpur, Federal Territory of Kuala Lumpur Malaysia

**Keywords:** Menopause, Reliability, Validity, Malay, Exploratory, Confirmatory

## Abstract

**Background:**

The Menopause Rating Scale (MRS) is an internationally used tool to measure menopause-related symptoms and to date it is unavailable in the Malay language. We aimed to translate and validate the Malay language version of the MRS.

**Methods:**

Translation of the English version of MRS into Malay was done by a bilingual expert and back translated. Translated version of MRS was reviewed by a panel to determine the face validity. A sample of 321 women aged 40–60 years residing in Klang, Selangor, Malaysia was selected by stratified random sampling method in a house-to-house survey. The Malay language version of MRS was self-administered. Reliability analyses, including test–retest reliability (on 30 women after a two-week interval) were conducted. To ascertain the construct validity, 11 items were analyzed confirmatory factor analysis was conducted to evaluate the structural model fit of the Malay language version of MRS.

**Results:**

A total of 294 (91.6%) completed the survey and their mean age was 50.9 years (SD = 6.3). An overall Cronbach’s alpha for MRS was 0.904. Cronbach’s alpha for psychosomatic, urogenital, and somatovegetative subscales were 0.889, 0.846, and 0.776 respectively. The corrected item correlations were approximately 0.6 and inter-item correlations were between 0.3 and 0.9. On exploratory structural equation modelling, the chi-square test of goodness of fit yielded a significant value; χ^2^** = **78.4, *df* = 25, *p* < 0.001, (reported if N > 200). Additionally, the value of Tucker–Lewis Index (TLI) = 0.954showed a good fit to the model.

**Conclusion:**

The translated English version of the Menopause Rating Scale into the Malay language showed excellent reliability, test–retest reliability, and construct validity. The instrument can be used to assess menopause-related symptoms among Malaysian women.

**Supplementary Information:**

The online version contains supplementary material available at 10.1186/s12905-022-01922-8.

## Background

Menopause is a natural stage of biological changes in every woman’s life with the cessation of menstruation [1]. Declining hormones result in a range of symptoms women experiences during the period of menopause [2]. For the domains of physical, psychological, and urogenital symptoms, the frequency of occurrence and intensity of menopausal symptoms varies across geographical regions [3]. Such variations can be attributed to individual perceptions and psychosocial and cultural factors [4]. Nevertheless, menopausal symptoms are known to affect Health-related quality of life [5]. As life expectancy increases, the absolute number of women surviving into the perimenopausal, and postmenopausal phases increases [6]. This emphasizes the importance of healthcare providers using reliable and valid tools to assess menopausal symptoms and their severity to provide advice on how to cope with menopausal symptoms and referral services to those in need [7].

Several measurement scales have been developed to measure menopausal symptoms since the 1990s [8]. Among them, the Greene Climacteric Scale [9], the Menopausal Symptoms List [10], the Women’s Health Questionnaire [11], and the Menopause Rating Scale (MRS) [12] have gained widespread acceptance and use throughout the world. The MRS was developed in the early 1990s in the German language [5]. MRS a self-administered questionnaire to assess the frequency and severity of menopausal symptoms among both peri-and post-menopausal women [13]. MRS questionnaire has been used by physicians to assess climacteric symptoms after treatment. MRS has fared well in relation to other questionnaires (α = 0.91) making it a reliable and valid questionnaire for measuring the menopausal quality of life and routine evaluation of menopausal symptoms [14]. Following the initial translations from German to English, which demonstrated superior psychometric properties, the English version of MRS has been translated and validated into approximately 30 international languages [14]. Cronbach's alpha indicated overall reliability of 0.83 in several countries [15]. MRS versions in Serbian [16], traditional Chinese [17], Urdu [18], Persian [19], and Indonesian Bahasa [20] have demonstrated acceptable reliability and validity.

Menopausal symptoms are usually considered as a taboo and sensitive among Malaysian women. They are often reluctant to discuss about menopausal symptoms and tend to suffer in silence [21]. Thus there is a need to improve women’s awareness about menopausal symptoms. To address this issue menopausal symptoms in Malaysian women have been studied [22, 23]. Malaysian studies have used MRS to interview the women face-to-face in Malay language, which is a national language of a multiethnic Malaysian population. There is a need for a valid, self-administered questionnaire to assess women’s experience of menopausal symptoms. A study from Sarawak in east Malaysia reported an acceptable reliability of the questionnaire in Malay language version of MRS that was modified to provide dichotomous (yes/no) response options for MRS instead of rating their severity. However, a study from Indonesia that translated original MRS into Bahasa Indonesian language has reported an acceptable construct validity [20]. Adopting MRS into the Malay language would enable the identification and assessment of menopause-related symptoms among Malaysian women, as well as to make comparisons of prevalence and severity of menopausal symptoms among women in other countries [15]. It would also allow comparisons between the occurrence and severity of menopause symptoms pre-and post-treatment with hormone replacement therapy, non-hormonal therapy, or supplements [24]. Nevertheless, to-date none of the Malaysian studies have tested the validity of self-administered Malay language version of MRS. To fill this gap, we aimed to translate the MRS from English into Malay language, as well as to assess its reliability and construct validity.

## Methods

### Design, setting, and participants

A cross-sectional community-based self-administered interview study was conducted in Klang, Selangor Malaysia**.** Klang is a district in Selangor. Klang has a mixed population of Malay and, Chinese, Indians, and other indigenous ethnicities. Malaysian women between the ages of 40 and 60 who resided in Klang were eligible to participate. Women who had attained artificial menopauses (either medical or surgical), on hormone replacement therapy, pregnant/lactating, who had heart ailments, psychiatric conditions, history of drug or alcohol abuse, on cancer treatment, and those with premature ovarian failure or genital malformation were excluded.

### Sample size and sampling method

The sample size was calculated for 95% confidence limits (Z = 1.96), an allowable error of 5%, and an anticipated proportion of 80% for the presence of menopausal symptoms. The minimum sample size was 249 using the formula, and after allowing for a 20% non-response rate, the final sample size was 294 using the formula. A minimum sample of 200 is adequate to perform validation of the questionnaire for meaningful and interpretable values [25]. Three streets in Klang (Taman Petaling, Southern Park, and Taman Chi Liung) were chosen at random from a list of all streets. From a total of 58 rows of houses in these three streets, 14 rows were chosen by simple random sampling. From each row of houses, it was decided to visit consecutive households until seven eligible women were recruited. Thus, 98 houses were selected from each street to recruit 294 women. Each eligible household member was invited to participate in this study.

### Instrument

#### Menopausal Rating Scale

The MRS (Menopausal Rating Scale) is divided into three domains: (1) somato-vegetative, (2) psychological, and (3) urogenital [13]. (1) Somatovegetative domain: Hot flushes, heart discomfort, sleep problem and muscles, and joint problems. (2) Psychological domain: depression, irritability, anxiety, and physical and mental exhaustion (3) Urogenital domain: Sexual problems, bladder problems, and dryness of the vagina. A five-point scale of severity is used to rate each symptom. Each item can be graded from 0 to 4, (0 = not present), (1 = mild), (2 = moderate), (3 = severe), (4 = very severe). The scores of the Somatovegetative domain range from 0 to 16, the urogenital domain 0 to 12, and the psychological domain 0 to 16. The composite score ranges from 0 to 44 [26].

#### Translation Process

The self-administered original menopause rating scale in English was translated into Malay language using forward and backward translation methods. The forward translation was conducted by a bilingual expert. The translated Malay language version was again reviewed by the researcher who is also proficient in Malay language and conversant with the Malaysian socio-cultural context. A blind bilingual expert back translated the Malay version into the English language. Following this all translators created a final, consolidated version and approved the final version. The final translated Malay language version was reviewed by a panel of health educators, a gynecologist, and a family medicine expert familiar with dealing with women of menopausal and postmenopausal age. The panel members were tasked with reviewing and evaluating the items, as well as determining their relevance and appropriateness for the Malaysian context. Finalized version was pretested during a pilot survey conducted among 30 Malay speaking women attending the outpatient clinics, who were specifically instructed to provide feedback. Malay languag and the original English versions are provided in Additional file 1 (Malay and English language versions of MRS).

#### Data collection procedures

The respondents were chosen using the sampling procedure through a house-to-house survey during the March and April 2020. Each woman who met the criteria for participation was invited to take part. The purpose of the survey was explained, anonymity and confidentiality were assured, and consent was sought for participation. Participants were provided with a study/participant information sheet, and participants were allowed to clarify on the questionnaire. The questionnaire was distributed to the consenting women and a completed form were collected. Consent was sought to complete the same questionnaire after an interval of two weeks. Thirty eligible women were recruited for test–retest. The study was approved by the International Medical University Ethics Joint committee. The project identification number MSPH 1- 2019 (01).

### Statistical analyses

Data was analyzed using Statistical Package for Social Sciences (SPSS) version 25. Descriptive statistics were computed. The mean, standard deviation, range, skewness, and kurtosis for the scores of the questionnaire were computed. Normality of data distribution was tested by one-sample Kolmogorov–Smirnov tests. Cronbach’s alpha, scale variance if item deleted, inter-item correlation, and corrected item-total correlation, were estimated for reliability analysis. Cronbach’s alpha measures internal consistency and a value greater than 0.7 indicates adequate internal consistency [27]. For the test–retest validity, we estimated to examine the test–retest reliability Pearson’s correlation coefficients and intraclass correlation coefficients (ICC) were estimated and a value greater than 0.75 indicates stability or acceptable test–retest reliability [28].

In previous research on translated versions of MRS, confirmatory factor analysis has encountered “straddling" with the factor structure of MRS [17, 19, 29]. To establish the construct validity of Malay language version of MRS, Exploratory Structural Equation Modelling (ESEM) is the recommended statistical approach which integrates the best features of exploratory and confirmatory factor analyses by allowing cross-loadings of items in the analyses while taking on the features of structural equation modelling [30]. ESEM was done on JASP software.

## Results

### Descriptive statistics

A total of 321 eligible participants were invited to participate, of whom 27 declined, giving a response rate of 91.6%. Sociodemographic characteristics of the respondents are shown in Table 1. The mean age for respondents is 50.93 (SD = 6.25) years and approximately 57% of them were aged between 50 and 60 years. All three ethnic groups namely Malays (35.4%), Indians (33.7%) and Chinese (31.0%) were equitably represented. The majority (> 80%) were educated at a secondary level and were currently married. Nearly a third of them were housewives or retired and another third were professionals (Nurse, teachers, lawyers, etc.). The three most frequently reported symptoms were ‘sleep problems’ 111 (37.8%), ‘physical and mental exhaustion 110 (37.4%), and sexual problems 106 (36.1%) which were rated as ‘moderate’, ‘mild’ and ‘moderate’ respectively in terms of severity.

**Table 1:** Sociodemographic characteristics of the survey participants

	Number (%)
Age	
40–44 years	56 (19.0)
45–49 years	69 (23.5)
50–54 years	63 (21.4)
55–60 years	106 (36.1)
Race	
Malay	104 (35.4)
Indian	99 (33.7)
Chinese	91 (31.0)
Education	
Primary	51 (17.3)
Secondary	133 (45.2)
Tertiary	110 (37.4)
Employment categories	
Housewife/retired	104 (35.4)
Business	54 (18.4)
Professional	100 (34.0)
Other jobs	36 (12.2)
Average family income	
< RM 3000	116 (39.5)
RM3000-RM 5000	105 (35.7)
RM5000-RM 8000	50 (17.0)
RM8000 and above	23 (7.8)
Marital status	
Married	261 (88.8)
Separated/divorce	14 (4.8)
Single	19 (6.5)

The average scores of MRS Malay items and sub-scales are shown in Table 2. The overall mean MRS score was 13.7 (8.7) and the means for subscales were 5.2, (4.0), 5.1 (3.3), and 3.6 (2.8) for psychological, Somatovegetative, and urogenital domains respectively. Among the MRS items ‘hot flushes’, ‘physical exhaustion and sleep problems’ scored the highest (Table 2).

**Table 2:** Descriptive statistics of the 11 items of the Malay language version of MRS

Items (Bahasa Melayu)	Item (English)	Mean	Standard deviation	Skewness	Kurtosis
Rasa panas, berpeluh	Sweating/flushes	1.64	1.14	0.076	− 0.0841
Ketidakselesaan jantung	Heart discomfort	0.35	0.72	2.138	4.169
Masalah tidur	Sleep problems	1.54	1.12	0.070	− 0.810
Mood kemurungan	Depressive mood	0.93	1.15	0.924	− 0.387
Cepat marah	Irritability	1.46	1.22	0.584	− 0.588
Kebimbangan	Anxiety	1.13	1.23	0.724	− 0.689
Keletihan fizikal dan mental	Exhaustion	1.69	1.02	0.352	− 0.355
Masalah seksual	Sexual problems	1.41	1.13	0.100	− 1.018
Masalah pundi kencing	Bladder problems	0.72	0.98	1.095	− 0.064
Kekeringan vagina	Vaginal dryness	1.42	1.14	0.119	− 1.095
Ketidakselesaan sendi dan otot	Joint and muscle discomfort	1.51	1.20	0.295	− 1.074

### Reliability statistics

The item-wise reliability statistics are shown in Table 2 whereas the overall and subscale reliability statistics along with test–retest reliability are shown in Table 3. An overall Cronbach’s alpha for MRS-BM was 0.904 indicating that it was appropriate as a Malay translation of MRS. However, Cronbach’s alpha was slightly lower for urogenital and Somatovegetative subscales than psychosomatic (Table 3). The corrected item-total correlations were beyond 0.60 except for two items namely ‘heart discomfort’ (0.37) and ‘bladder problems’ (0.51) which is higher than recommended threshold 0.30 [28] and inter-item correlations were within an acceptable range 0.30–0.90 [31]. In addition, the Cronbach Alpha if Item Deleted values were about 0.9 for all items indicating that all items can be retained (data not shown). Correlations between the initial test–retest ranged from moderate to strong but the intra-class correlation was very high (0.88–0.98) indicating a high concordance for the responses given by the respondents at two-week intervals. In addition, the subscales had close correlations with their own scales rather than with other domains.

**Table 3:** Reliability statistics of of MRS Malay language version

Dimension	Cronbach’s alpha	Range of inter-item correlation	Test–retest reliability
			Intra-class correlation coefficient (95% CI)
Psychological (4, 5, 6 & 7)	0.889	0.577–0.776	0.941 (0.877–0.972)
Somatovegetative (1, 2, 3 & 11)	0.776	0.248–0.666	0.974 (0.946–0.988)
Urogenital (8, 9 & 10)	0.846	0.485–0.899	0.959 (0.916–0.981)
Overall	0.904		0.968 (0.933–0.985)

### Exploratory structural equation modelling

On ESEM, the Chi Square Test of Goodness of Fit yielded a significant value χ^2^ = 78.4, *df* = 25, *p* < 0.001, which was below the threshold of 0.05 (reported if N > 200). Additionally, root mean square error of approximation (RMSEA) = 0.081 and Tucker–Lewis Index (TLI) = 0.954 shows a good model fit to the overall model. No items were discarded as all items are beyond 0.50 factor loading.

## Discussion

The MRS originally developed in German and later translated into English was used to translate and validated into Malay language. The survey, which utilized the Malay language version of MRS was administered to a representative sample of Malaysia's multiethnic population, demonstrated acceptable psychometric properties for assessing menopausal symptoms in Malaysian women. The Malay language version of MRS demonstrated high content validity, as well as an appropriate model structure and fit. The reliability index including repeatability was also high. Malaysian women expressed no concerns about the ambiguous words or phrases Malay language version of MRS.

This study confirms that the overall reliability of the Malay language version of MRS was as good as previously reported, with a Cronbach's alpha of greater than 0.80 [16–20]. However, in a multi-national methodological study of MRS, Heinman et al. reported a Cronbach's alpha range of 0.60 to 0.90 [15]. Comparable reliability statistics were reported for Persian, Urdu, and Indonesian Bahasa versions conducted in similar socio-cultural contexts [18–20]. A relatively lower Cronbach alpha for urogenital and somatic compared to the psychological domain was observed in studies on validation of Chinese, Serbian and Urdu MRS versions [16–18]. However, in a validation study of the Indonesian Bahasa version, the CFA for all domains was consistently above 0.90 [20].

As with the Czech version [29], only two factors were loaded in this study, as opposed to the three factors in the original version, namely psychological, Somatovegetative, and urogenital. Since MRS is a tool that is easy to administer for clinical decision-making about an alternative diagnostic or treatment approach in outpatient settings we maintain three factors for confirmatory factor analyses [24]. However, the structure of the MRS questionnaire differs in this study as well. For instance, sexual problems and vaginal dryness were classified as somatic, whereas heart problems were classified as urogenital (data not shown). In the validation studies conducted on the Czech, Chinese, and Persian versions of MRS, this loading of domain items was observed to be different from the original version [17, 19, 29]. Such instability in factor structure was noted in a multi-country evaluation study by the authors of the original MRS questionnaire [15]. The authors attributed possible moderate correlations between the different domains of MRS since the 11 items of MRS are not independent of each other [15]. It has been well documented that menopause symptoms vary across countries and cultures based on the women’s perception of the symptoms.

The validated Malay language version of MRS is comparable in terms of reliability and validity to the Indonesian version. Indonesian version had much higher internal consistency overall and for individual domains (Cronbachs Alpha > 0.90). [20] In the Indonesian version, the three-factor model structure was adequate, but in the Malay language version of MRS, some items loaded under different domains on confirmatory factor analyses. Such differences may be attributed to the different socio-cultural milieu of the multi-ethnic Malaysian population. Though the Malaysian national language is Malay language, Indian original Malaysians mainly speak Tamil and the Chinese origin Malaysians speaks Mandarin, and the three ethnic groups are socioculturally distinct. Such differences may be attributed to the multi-ethnic Malaysian population's diverse socio-cultural milieu, with the major and other minority groups speaking in their preferred dialects even though Malay being the national language*.* Despite similarities. the Malay Language spoken in Malaysia and Indonesia is distinct in terms of spelling, grammar, pronunciation, and vocabulary. As a result, a distinct Malay language version of MRS is justifiable.

## Limitations

This study used a representative sample that included members of Malaysia's three major ethnic groups. The sample was drawn from a suburban community. The findings may have to be confirmed in other populations such as rural and indigenous communities. The Malay language version of MRS was self-administered, and self-reported symptoms weeks prior are subject to recall bias. The emotional status and circumstance would have resulted in misreporting. Misconceptions of sexuality and perceptions of the symptoms such as ‘hot flushes’ in hot and humid weather conditions prevailing in Malaysia.

## Conclusion

The Malay language version of MRS version has excellent reliability and construct validity. The Malay language version has a potential for application in clinical and research settings to assess menopausal symptoms among Malaysian women (Additional file 1).

## Supplementary Information

**Additional file 1.** Malay and English language versions of MRS.

## Data Availability

The datasets used and/or analyzed during the current study are available from the corresponding author on reasonable request.

## Abbreviations

MRS: Menopause Rating Scale
ESEM: Exploratory structural equation modelling
TLI: Tucker–Lewis Index
EFA: Exploratory factor analysis
CFA: Confirmatory factor analysis
