# Supplementary material for: Menopause Rating Scale (MRS) in the Malay language-translation and validation in a multiethnic population of Selangor, Malaysia
Source: BMC Womens Health. 2022 Aug 17;22:347. doi: 10.1186/s12905-022-01922-8 (PMC9387051; doi:10.1186/s12905-022-01922-8)
Supplement: Supplementary file 1 — Additional file 1. Malay and English language versions of MRS. [file 12905_2022_1922_MOESM1_ESM.pdf]

Appendix 4: Menopause Rating Scale Questionnaire in Malay

Menopause Rating Scale (MRS)

Mana satu di antara tanda-tanda berikut berlaku kepada anda pada masa ini? Sila, tandakan kotak yang sesuai bagi setiap simptom. Untuk gejala yang tidak berlaku, sila tandakan 'tiada'.

**Gejala:**	**tiada**	**sedikit**	**sederhana**	**teruk**	**sangat teruk**
Skor =	0	1	2	3	4
1.Rasa panas, berpeluh (Episod berpeluh)...	☐	☐	☐	☐	☐
2.Ketidakselesaan jantung (kesedaran luar biasa denyutan jantung, jantung melompat, lumba jantung, sesak)	☐	☐	☐	☐	☐
3.Masalah tidur (kesukaran untuk tidur, kesukaran untuk tidur berterusan, bangun awal)	☐	☐	☐	☐	☐
4.Mood kemurungan (rasa sedih, sedih,di ambang air mata, kekurangan semangat, perubahan emosi)………………….	☐	☐	☐	☐	☐
5. Cepat marah (rasa saraf, ketegangan dalaman, perasaan agresif)	☐	☐	☐	☐	☐
6.Kebimbangan (kegelisahan dalaman, rasa panik)	☐	☐	☐	☐	☐
7.Keletihan fizikal dan mental (penurunan umum dalam prestasi, memori terjejas, pengurangan dalam menumpukan perhatian,terlupa)………………………………..	☐	☐	☐	☐	☐
8.Masalah seksual (berubah dalam keinginan seks, dalam aktiviti seksual dan kepuasan)…………………………………	☐	☐	☐	☐	☐
9.Masalah pundi kencing (kesukaran dalam membuang air kecil, meningkatkan keperluan untuk membuang air kecil, pundi kencing inkontinen)…………………………………………	☐	☐	☐	☐	☐
10.Kekeringan vagina (sensasi kekeringan atau kesakitan dalam faraj, masalah dengan hubungan seks)	☐	☐	☐	☐	☐
11.Ketidakselesaan sendi dan otot (sakit pada sendi, aduan rheumatoid)	☐	☐	☐	☐	☐

Menopause Rating Scale (MRS)

Which of the following symptoms apply to you at this time? Please, mark the appropriate box for each symptom. For symptoms that do not apply, please mark 'none'.

**Symptoms:**	none	mild	moderate	severe	very severe
**Score =**	**0**	**1**	**2**	**3**	**4**
1. Hot flushes, sweating (episodes of sweating)	☐	☐	☐	☐	☐
2. Heart discomfort (unusual awareness of heart beat, heart skipping, heart racing, tightness)	☐	☐	☐	☐	☐
3. Sleep problems (difficulty in falling asleep, difficulty in sleeping through, waking up early)	☐	☐	☐	☐	☐
4. Depressive mood (feeling down, sad, on the verge of tears, lack of drive, mood swings)	☐	☐	☐	☐	☐
5. Irritability (feeling nervous, inner tension, feeling aggressive)	☐	☐	☐	☐	☐
6. Anxiety (inner restlessness, feeling panicky)	☐	☐	☐	☐	☐
7. Physical and mental exhaustion (general decrease in performance, impaired memory, decrease in concentration, forgetfulness)	☐	☐	☐	☐	☐
8. Sexual problems (change in sexual desire, in sexual activity and satisfaction)	☐	☐	☐	☐	☐
9. Bladder problems (difficulty in urinating, increased need to urinate, bladder incontinence)	☐	☐	☐	☐	☐
10. Dryness of vagina (sensation of dryness or burning in the vagina, difficulty with sexual intercourse)	☐	☐	☐	☐	☐
11. Joint and muscular discomfort (pain in the joints, rheumatoid complaints)	☐	☐	☐	☐	☐
